# Effect of tea polyphenols supplement on growth performance, antioxidation, and gut microbiota in squabs

**DOI:** 10.3389/fmicb.2023.1329036

**Published:** 2024-01-15

**Authors:** Ailing Chen, Tingting Ma, Yajing Zhong, Shan Deng, Shaoping Zhu, Zhiqi Fu, Yanhua Huang, Jing Fu

**Affiliations:** ^1^Innovative Institute of Animal Healthy Breeding, Zhongkai University of Agriculture and Engineering, Guangzhou, Guangdong, China; ^2^College of Animal Science and Technology, Zhongkai University of Agriculture and Engineering, Guangzhou, Guangdong, China; ^3^Guangdong Laboratory for Lingnan Modern Agriculture, Guangzhou, Guangdong, China

**Keywords:** tea polyphenols, squabs, growth performance, antioxidant capacity, intestinal barrier function

## Abstract

Early life nutritional supplementation can significantly improve pigeon health. Both the nutritional crops of parental pigeons and the intestinal development of squabs play key roles in the growth rate of squabs. Tea polyphenols (TPs), as natural plant extracts, exhibit potential biological activities. However, the impact of TPs on the intestinal function of squabs is not known. This study evaluated the effects of TPs on growth performance, immunity, antioxidation, and intestinal function in squabs. A total of 432 young pigeons (1 day old) were divided into four groups: a control group (fed a basic diet) and three treatment groups (low, medium, and high dose groups; 100, 200, and 400 mg/kg TPs, respectively). On the 28th day, samples of serum, mucosal tissue, and digests from the ileum of squabs were collected for analysis. The results revealed that TP supplementation significantly reduced the feed-to-meat ratio and improved the feed utilization rate and serum biochemical indices in squabs. Additionally, it enhanced the intestinal barrier function of birds by promoting intestinal development and integrity of tight junctions and regulating digestive enzyme activities and intestinal flora. Mechanistically, TPs activated the Nrf2-ARE signaling pathway, which may be associated with improved antioxidant and immune responses, correlating with an increased abundance of *Candida arthritis* and *Corynebacterium* in the ileum.

## 1 Introduction

Meat-type pigeons have been consumed in many countries for centuries because of their high nutritional value (Yin et al., 2022). In China, pigeons are raised as poultry to meet the growing demand for pigeon meat. Pigeons are altricial birds, differing from precocial species such as chickens and geese in terms of nesting instinct, rearing offspring, and growth and development. Squabs cannot fend for themselves and become market-ready at about 500 g (about 25 days old) when they leave the nest. Therefore, male and female pigeons regurgitate nutrition to squabs after hatching. This gradual process, similar to piglet weaning, is also termed “weaning” for pigeons (Wen et al., 2022). Xu et al. (2022) demonstrated that the growth rate of squabs is closely related to the intestinal absorption capacity of nutrients. This indicates that the nutritional crops of parental pigeons and the intestinal development of squabs are crucial to the growth rate of squabs.

Tea polyphenols (TPs) are extracted from tea, comprising flavonols, anthocyanins, flavonoids, and phenolic acids. TPs have many effects, such as regulating intestinal flora and anti-oxidation, with catechin being the main component (Su et al., 2019; Shao et al., 2022). Epigallocatechin-3-gallate (EGCG) is the most abundant and effective active ingredient in catechins (Khan and Mukhtar, 2018). TPs, as natural antioxidants, enhance animal antioxidant capacity by scavenging reactive oxide species and boosting antioxidant enzyme activities. They have been shown to improve the activities of catalase (CAT), superoxide dismutase (SOD), and glutathione peroxidase (GSH-Px), offering antioxidant benefits (Mao et al., 2017; Yan et al., 2020). Furthermore, TPs promote beneficial intestinal bacteria like bifidobacteria and short-chain fatty acids (SCFAs) production. This acidification of the intestine aids in mineral absorption, inhibits pathogenic bacterial growth, and contributes to intestinal health (Sun et al., 2018). Xue et al. (2017) reported that dietary supplementation with EGCG significantly increased body weight and feed intake of broilers and enhanced broiler appetite under heat stress to improve growth performance.

Previously, we reported that TPs improved egg quality and maintained homeostasis in parental pigeons (Chen et al., 2022). Hence, in this study, we further explored the effects of TPs on growth performance, anti-oxidation, and immune and intestinal barrier functions in offspring pigeons. Currently, there are very limited studies examining the influence of TPs on pigeon intestinal flora. Our results provide new insights for using TPs to enhance pigeon quality and economic returns. Our results suggest the potential health benefits of a TP-based nutritional plan.

## 2 Materials and methods

TPs (purity >98.0%) used in the study were obtained from Chengdu Wagott Bio-tech Co., Ltd. (China).

### 2.1 Animals, treatments, and ethics statement

In total, 432 squabs were used. These 1-day-old squabs were hybrid offspring of White King pigeons and Shenwang pigeons. The squabs were randomly divided into four groups, with each treatment having six replicates, and each replicate included 18 squabs. The C (control) group was provided with the basic diet, while the other three treatment groups, named TL, TM, and TH for low, medium, and high, had 100, 200, and 400 mg/kg of TPs added to their diets, respectively. Each iron cage housed two squabs along with their parental pigeons (male and female pigeons). The cages were equipped with a nest, a feed tank, and a water tank to provide suitable conditions. To minimize the potential impact of cage location, the replicates were evenly distributed across the upper, middle, and lower levels of the cages. During the whole experiment, young breeding pigeons were provided with *ad libitum* feed, water, and health care. Young breeding pigeons fed these squabs with crop milk in a beak-to-beak manner until the 28th day of age. The basic diet was prepared according to the NRC (Nutrient requirements of poultry. Washington, DC: The National Academies Press, 1994) standard with a ratio of “30% pellet feed + 70% raw feed” according to the feeding habits and production practices of meat pigeons. Also, we maintained 16 h of light every day, a combination of natural and artificial light. In this study, there was a 7-day pre-feeding period followed by a 28-day breeding period. The composition and nutrient levels of both the pelleted feed and raw grain of the basic diet (on an air-dry basis) are provided in Tables 1, 2.

**TABLE 1:** Composition and nutrient levels of the raw grain in basic diet (air-dry basis).

Ingredients	Content (%)	Nutrient levels	Content (%)
Corn	37.50	ME/(MJ/kg)	13.96
Sorghum	31.25	CP	13.82
Wheat	15.625	EE	2.65
Peas	15.625	Ca	0.26
Total	100	TP	0.30
		Lys	0.67
		Met	0.15
		Thr	0.50
		Val	0.66
		Ile	0.53
		Phe	0.64

ME was a calculated value, while other indicators were measured values.

**TABLE 2:** Composition and nutrient levels of the pelleted feed in basic diet (air-dry basis).

Ingredients	Content (%)	Nutrient levels^2^	Content (%)
Corn	40.00	ME/(MJ/kg)	11.10
Wheat bran	4.18	CP	20.87
Wheat middling	10.00	EE	4.26
Soybean mea	32.50	Ca	0.91
Rice bran meal	5.00	TP	0.56
Soybean oil	4.00	Lys	1.05
Premix^1^	0.55	Met	0.24
CaHPO_4_	0.59	Thr	0.78
Limestone	1.73	Val	0.99
Lys (98.5%)	0.12	Ile	0.84
Met (99%)	0.12	Phe	0.95
NaHCO_3_	0.08		
Choline chloride (60%)	0.08		
Antifungal agent	0.10		
NaCl	0.20		
Bentonite	0.75		

^1^The premix provided the following per kilogram of the diet: VA 4 000 IU, VE 20 IU, VB1 5 mg, VB2 8 mg, VB6 4 mg, VB12 5 ug, nicotinic acid 20 mg, folic acid 0.4 mg, D-pantothenic acid 20 mg, biotin 0.15 mg, choline chloride 450 mg, Cu 5 mg, Fe 35 mg, Mn 50 mg, Zn 55 mg, I 0.2 mg, Se 0.2 mg.

^2^ME was a calculated value, while other indicators were measured values.

### 2.2 Sample collection and preparation

At the end of the experiment, 2 squabs were randomly selected per replicate (a total of 12 squabs for each group) and sacrificed for postmortem analysis. Before this, the squabs were fasted overnight (12 h), and blood samples were collected from the necks of the squabs. The separated serum samples were stored at −80°C for subsequent analysis of blood biochemical, antioxidant, and immune indices. The squabs were subsequently slaughtered, and their ileum tissues were collected. Some of the intestinal tissues were fixed in a 4% paraformaldehyde solution for morphological observation. The remaining part was stored at −80°C for the detection of intestinal antioxidant indices, immune indices, tight junction proteins, and mRNA expressions of antioxidant-related genes. Additionally, the ileum contents of the squabs were collected and stored at −80°C for the analysis of intestinal digestive enzyme activity and microbial diversity.

### 2.3 Growth performance parameters

The weekly feed intake of the squabs was recorded for each repetition throughout the experiment. Additionally, the squabs were weighed on an empty stomach at specific ages (1, 7, 14, 21, and 28 days). When the squabs were 1 and 28 days old, the young breeding pigeons were weighed on an empty stomach by the nest. After the experiment, the average daily gain (ADG) of squabs, the weight loss of breeding pigeons, the average daily feed intake, and the nest feed conversion ratio were calculated in each replicate as follows:

$$\mathrm{ADG}\,\mathrm{of}\,\mathrm{squabs}\,\left(g/d\right)=\frac{\mathrm{Final}\,\mathrm{weight}-\mathrm{Initial}\,\mathrm{weight}}{\mathrm{Test}\,\mathrm{days}}$$

$$\begin{array}{l} \mathrm{Weight}\,\mathrm{loss}\,\mathrm{of}\,\mathrm{breeding}\,\mathrm{pigeons}\,\left(g\right)\\ \;=\mathrm{Final}\,\mathrm{weight}\,\mathrm{of}\,\mathrm{breeding}\,\mathrm{pigeons}\\ \;-\mathrm{Initial}\,\mathrm{weight}\,\mathrm{of}\,\mathrm{breeding}\,\mathrm{pigeons} \end{array}$$

$$\begin{array}{l} \mathrm{Average}\,\mathrm{daily}\,\mathrm{feed}\,\mathrm{intake}\,\left(g/d\right)\\ \;=\frac{\mathrm{Total}\,\mathrm{feed}\,\mathrm{intake}\,\mathrm{per}\,\mathrm{nest}\,\mathrm{of}\,\mathrm{pigeons}}{\mathrm{Test}\,\mathrm{days}} \end{array}$$

$$\begin{array}{l} \mathrm{Nest}\,\mathrm{feed}\,\mathrm{conversion}\,\mathrm{ratio}\\ \;=\frac{\mathrm{Total}\,\mathrm{feed}\,\mathrm{intake}\,\mathrm{per}\,\mathrm{nest}\,\mathrm{of}\,\mathrm{pigeons}}{\begin{array}{c} \mathrm{Final}\,\mathrm{weight}\,\mathrm{of}\,\mathrm{breeding}\,\mathrm{pigeons}\,\mathrm{and}\,\mathrm{squabs}\\ \;-\mathrm{Initial}\,\mathrm{weight}\,\mathrm{of}\,\mathrm{breeding}\,\mathrm{pigeons}\,\mathrm{and}\,\mathrm{squabs} \end{array}} \end{array}$$

### 2.4 Serum biochemical indices

The blood biochemical indices of squabs were detected by the HITACHI 7180 automatic biochemical analyzer (Japan), and the corresponding kits were purchased from Mike Biology Co., Ltd. (China). Serum biochemical indices included total protein (TP), albumin (ALB), total cholesterol (TC), triglyceride (TG), glucose (GLU), low-density lipoprotein (LDL) and high-density lipoprotein (HDL), aspartate aminotransferase (AST), alanine aminotransferase (ALT), alkaline phosphatase (ALP), and globulin (GLO).

### 2.5 Antioxidant parameters in serum and ileum

The total antioxidant capacity (T-AOC), the activities of total superoxide dismutase (T-SOD), GSH-Px, and lactate dehydrogenase (LDH), and the concentration of malondialdehyde (MDA) in the serum and ileum of squabs were measured with corresponding assay kits (Nanjing Jiancheng Bioengineering Institute, Jiangsu, China).

### 2.6 Enzyme-linked immunosorbent assay

The concentrations of secretory IgA (sIgA), interleukin 1β (IL-1β), IL-2, IL-10, and tumor necrosis factor-alpha (TNF-α) in ileum segments of squabs were determined using corresponding ELISA kits at 450 nm (Quanzhou Kenuodi Biology Technology Co., Ltd., Fujian, China) according to the manufacturer’s instructions. Also, the serum concentrations of immunoglobulin A (IgA), IgM, and IgG were measured. Cytokine concentrations were calculated using the standard curve.

### 2.7 Intestinal morphology examination

Approximately 0.5 cm of ileum tissues were collected and fixed in a 4% paraformaldehyde solution, and processed in paraffin. The tissue sections (5–6 μm) were placed on the glass slides, stained with hematoxylin and eosin (H&E), and then observed under an optical microscope. The villous height, crypt depth, and villus height/crypt depth ratio were determined using the Image J software.

### 2.8 Quantitative real-time PCR

Total RNA from ileal mucosa was extracted by the TRIzol reagent, and its concentration and purity were measured by a NanoDrop One spectrophotometer (Thermo Fisher Scientific, USA). The extracted RNA was quickly reverse-transcribed into cDNA using TaKaRa reagent (Thermo Fisher Scientific, USA) according to the manufacturer’s instructions, and the obtained cDNA was stored as a template at −20 °C for later use. Real-time PCR was conducted using the CFX Connect fluorescent quantitative PCR system (BIO-RAD, USA) with PowerUp™ SYBR™ Green Master Mix (Thermo Fisher Scientific, USA) following the manufacturer’s instructions. Each sample had three parallels, and β-*actin* was used as the housekeeping gene. The specific primers used are listed in Table 3, which were designed using NCBI Primer-BLAST and synthesized by Shanghai Bioengineering Co., Ltd (China). The relative gene expression was calculated by the 2^–ΔΔCt^ method.

**TABLE 3:** Primers.

Genes	Primer (5′ to 3′)	Product size, bp	Genes number
β-*actin*	F:CCCATCTACGAAGGCTACGC R:CTTGATGTCACGCACAATTTC	149	XM_005504502.2
*Claudin2*	F:GTGCAGATGGGAACAAGGT R:GAGCCAAGGAAGCTACGG	119	XM_021283269.1
*Claudin3*	F:ACCTCATCCCCGTCTCCT R:CAGCCCACGTAGAGCGA	109	XM_005515008.2
*Occludin*	F:CAGGACGTGGCAGAGGA R:GTGGAAGAGCTTGTTGCGT	105	XM_005509325.2
*ZO-1*	F:GAACCAAAGCCAGTGTATG R:GGTCCCCTTCCTCTAATC	247	XM_021299314.1
*SOD1*	F:TAAAGGAGGTGTGGCGGAAG R:GGTCATCGCGTCTTTCATGG	107	XM_013370038.1
*SOD2*	F:GAGCAGGGACGCCTACAAAT R:GCCACAGGCACTACTACAAGA	282	XM_013368727.2
*CAT*	F:GGGACTATCCGTCCTGGTCT R:CCCACAGGGATGAGAGGGTA	127	XM_005511042.2
*HO-1*	F:CACTGTCCTGGGAGCACAAG R:ACATGCTTTCGGAGCTGTGT	120	XM_005502753.2
*Nrf2*	F:AATCATGCACTTCCCTGGGGT R:AAAACTTCACGCCTTGCCCC	157	XM_021281726.1
*Keap1*	F:CTCGCCCTCCAAGGATTACC R:AAGGATTGGCGGAAGTAGCC	129	XM_005515118.1

### 2.9 Digestive enzyme activity

The ileum contents of squabs, stored at −80 °C, were used to measure digestive enzyme activity. The tissue homogenates were prepared in ice-cold saline at a ratio of 9:1. The homogenates were centrifuged at 2500 rpm for 10 min, and the collected supernatant was detected for the activities of α-amylase, trypsin, and lipase (LPS) using kits from Nanjing Jiancheng Bioengineering Institute as per their instructions. Additionally, the total protein (TP) concentration of the ileum contents was measured by an assay kit.

### 2.10 16S rRNA sequencing analysis

The ileum contents of six squabs were randomly selected from each group. The total DNA of the microbial community was extracted according to the instructions of the E.Z.N.A.^®^ Soil DNA Kit (Omega Bio-Tek, USA). The integrity and quality of the extracted DNA were detected by 1% agarose gel electrophoresis (voltage, 5 V/cm; time, 20 min). The concentration and purity of DNA were determined by the NanoDrop 2000 ultramicro spectrophotometer. The primers 338F (5′-ACTCCTACGGGGAGGCAGCAG-3′) and 806R (5′-GGACTACHVGGGTWTCTAAT-3′) were used to amplify the conserved region of the V3-V4 region of the 16S rRNA gene. The amplified PCR products were examined by 2% agarose gel electrophoresis. The PCR products were purified using the AxyPrep DNA Gel Extraction Kit (Axygen Biosciences, USA). The recovered products were quantified using a Quantus™ Fluorometer (Promega, USA) following the manufacturer’s instructions. Purified amplicons were pooled in an equimolar ratio and subjected to paired-end sequencing on an Illumina MiSeq PE300 platform by Majorbio Bio-Pharm Technology Co. Ltd (Shanghai, China).

### 2.11 Statistical analysis

In addition, the 16S rDNA high-throughput sequencing was analyzed using one-way ANOVA in SPSS 23.0. A Tukey’s test was used to compare the significant differences between the average values. Orthogonal polynomials determined the effects of different inclusion levels of dietary TPs for linear and quadratic effects. The results are expressed as the mean ± SEM. *P* < 0.05 indicates a significant difference, and *P* > 0.05 indicates no significant difference.

The raw 16S rRNA gene sequencing reads were demultiplexed, quality-filtered by fastp, and merged by FLASH. The operational taxonomy unit (OTU) clustering used UPARSE to cluster at a 97% similarity level for obtaining representative sequences. Each sequence was annotated using the RDP classifier database for species classification. The taxonomy of each sequence was analyzed using the RDP Classifier algorithm against the Silva 138. For the results from OTU cluster analysis, a Venn chart in R 3.3.1, the Tukey test for Alpha diversity index comparisons, and ANOSIM for PCA analysis were employed. These methods tested community structure differences between groups. Species relative abundance data in the sample were tested using the Wilcox rank sum test in Stats in R 3.3.1 and Scipy in Python. This test identified significant differences in the top 10 bacteria at the genus level based on relative abundance. The statistical results indicated that *P* < 0.05 signifies significance, and *P* > 0.05 does not. Finally, the coexistence relationship between species in the microbial community and environmental factors was analyzed using network analysis with the Spearman test. Species with *P* < 0.05 were highlighted by default.

## 3 Results

### 3.1 Effect of TPs on the growth performance of squabs

TP treatment groups, compared with the control (C) group, exhibited significantly lower nest feed conversion ratios (*P* < 0.05, Table 4). However, there was no significant effect on the initial and final weight and average daily feed intake of breeding pigeons and squabs (*P* > 0.05).

**TABLE 4:** Effects of tea polyphenols on growth performance of squabs.

Items	C group	TL group	TM group	TH group	ANOVA *P*-value	Linear *P*-value	Quadratic *P*-value
**Squabs**							
Initial weight (g)	19.36 ± 1.82	20.22 ± 1.56	18.56 ± 2.73	18.99 ± 2.37	0.297	0.367	0.888
Final weight (g)	466.90 ± 38.39	494.80 ± 36.36	477.30 ± 26.93	500.40 ± 27.93	0.059	0.048	0.817
ADG of squabs (g/d)	15.98 ± 1.32	16.95 ± 1.28	16.38 ± 1.00	17.19 ± 1.00	0.059	0.040	0.808
**Pair of breeding pigeon**							
Initial weight (g)	1067.40 ± 60.40	1084.60 ± 53.17	1138.40 ± 63.99	1081.60 ± 64.27	0.224	0.621	0.080
Final weight (g)	974.80 ± 49.72	983.40 ± 50.08	1047.60 ± 70.52	978.80 ± 30.41	0.079	0.745	0.035
Weight loss of breeding pigeons (g)	92.60 ± 17.59	101.20 ± 8.93	90.80 ± 41.13	102.80 ± 37.34	0.860	0.051	0.089
Average daily feed intake (g/d)	141.08 ± 14.02	132.95 ± 1.55	130.52 ± 3.82	131.23 ± 3.10	0.085	0.051	0.089
Nest feed conversion ratio	4.93 ± 0.45^a^	4.41 ± 0.29^b^	4.43 ± 0.17^b^	4.27 ± 0.05^b^	0.003	0.002	0.058

^a,b^Different superscripts in the same row denote significant differences (*P* < 0.05). Results were the Mean ± SD.

### 3.2 Serum biochemical indices

The health status of squabs was examined based on serum biochemical indices (Table 5). Compared with the control group, TP treatment groups exhibited a significant decrease in the activities of AST and ALT and the serum contents of TC in squabs (*P* < 0.05). The serum TP contents in the TL and TM groups were significantly increased by 25.97 and 37.59%, respectively (*P* < 0.05). The serum content of GLO was significantly increased in the TM group (*P* < 0.05), while the serum TG content was significantly decreased in the TH group (*P* < 0.05). However, the serum contents of ALB, GLU, LDL, HDL, and ALP activity showed no significant effect of TPs in squabs (*P* > 0.05).

**TABLE 5:** Effects of tea polyphenols on serum biochemical indexes of squabs.

Items	C group	TL group	TM group	TH group	ANOVA *P*-value	Linear *P*-value	Quadratic *P*-value
TP (g/L)	22.53 ± 4.35^b^	28.38 ± 1.18^a^	31.00 ± 4.05^a^	27.60 ± 3.58^ab^	0.004	0.044	0.001
ALB (g/L)	5.27 ± 0.92	5.48 ± 0.45	5.27 ± 0.93	5.07 ± 1.20	0.889	0.585	0.691
GLO (g/L)	17.27 ± 3.45^b^	22.9 ± 1.28^ab^	25.73 ± 4.81^a^	22.53 ± 3.55^ab^	0.004	0.033	0.002
AST (U/L)	142 ± 11.51^a^	117.6 ± 8.11^b^	106 ± 11.47^b^	112.8 ± 12.01^b^	<0.001	<0.001	<0.001
ALT (U/L)	33.6 ± 2.73^a^	21.6 ± 2.50^bc^	23.8 ± 2.86^b^	18.40 ± 1.85^c^	<0.001	<0.001	0.001
ALP (U/L)	549.00 ± 72.49	475.33 ± 143.9	436.17 ± 88.57	390.17 ± 139.65	0.141	0.028	0.499
TC (mmol/L)	9.00 ± 1.11^a^	7.32 ± 0.53^b^	7.39 ± 0.31^b^	7.33 ± 0.60^b^	0.001	0.003	0.005
TG (mmol/L)	2.15 ± 0.47^a^	1.6 ± 0.29^ab^	1.83 ± 0.20^ab^	1.50 ± 0.35^b^	0.020	0.014	0.403
GLU (mmol/L)	15.71 ± 1.79	12.01 ± 4.11	11.60 ± 2.44	13.16 ± 2.38	0.082	0.254	0.024
LDL (mmol/L)	3.84 ± 0.64	3.27 ± 0.83	3.35 ± 0.52	3.07 ± 0.49	0.223	0.078	0.482
HDL (mmol/L)	4.09 ± 0.25	4.30 ± 0.42	4.45 ± 0.66	4.58 ± 0.28	0.257	0.059	0.562

^a,b,c^Means with no common superscripts in the same row denote significant differences (*P* < 0.05). Results were the Mean ± SD (*n* = 6). TP, total protein; ALB, albumin; GLO, globulin; AST, aspartate aminotransferase; ALT, alanine aminotransferase; ALP, alkaline phosphatase; TC, total cholesterol; TG, triglyceride; GLU, glucose; LDL, low-density lipoprotein; HDL, high-density lipoprotein.

### 3.3 Oxidative parameters in the serum and ileum tissue

As listed in Table 6, serum GSH-Px activity was significantly higher (*P* < 0.05) and MDA content was significantly lower (*P* < 0.05) in squabs of TP treatment groups compared to the control group. Meanwhile, T-AOC was increased by 38.46% and 28.21% in the TL and TM groups, respectively, while T-SOD activity was increased in the TM and TH groups (*P* < 0.05). However, the serum LDH activity showed no significant difference in squabs among different groups (*P* > 0.05).

**TABLE 6:** Effects of tea polyphenols on serum antioxidant capacity of squabs.

Items	C group	TL group	TM group	TH group	ANOVA *P*-value	Linear *P*-value	Ouadratic *P*-value
T-AOC (mmol/L)	0.39 ± 0.06^b^	0.54 ± 0.10^a^	0.50 ± 0.05^a^	0.47 ± 0.04^ab^	0.008	0.244	0.005
T-SOD (U/ml)	241.17 ± 16.14^b^	261.55 ± 14.64^ab^	274.44 ± 7.52^a^	284.99 ± 18.30^a^	<0.001	<0.001	0.100
GSH-Px (U/ml)	406.52 ± 9.89^c^	424.36 ± 4.59^b^	428.99 ± 6.79^ab^	438.15 ± 7.10^a^	<0.001	<0.001	0.021
LDH (U/L)	905.83 ± 209.40	893.83 ± 212.86	870.17 ± 112.51	737.00 ± 180.25	0.378	0.102	0.588
MDA (nmol/mL)	5.69 ± 0.73^a^	4.60 ± 0.27^b^	4.64 ± 0.26^b^	3.21 ± 0.44^c^	<0.001	<0.001	0.889

^a,b,c^Means with no common superscripts in the same row denote significant differences (*P* < 0.05). Results were the Mean ± SD (*n* = 6).

As shown in Table 7, MDA content in the ileum of squabs was significantly reduced (*P* < 0.05) in all experimental groups compared to the C group. The activities of T-SOD and GSH-Px in the ileum of the TM and TH groups were significantly increased (*P* < 0.05), and the T-AOC in the ileum of the TL group was significantly increased (*P* < 0.05). However, the activity of LDH in the ileum of the TH group was significantly decreased (*P* < 0.05).

**TABLE 7:** Effect of tea polyphenols on the activity of antioxidant enzymes in the ileum of squabs.

Items	C group	TL group	TM group	TH group	ANOVA *P*-value	Linear *P*-value	Ouadratic *P*-value
T-AOC (mmol g-1)	0.10 ± 0.01^b^	0.13 ± 0.01^a^	0.12 ± 0.01^ab^	0.10 ± 0.02^b^	0.003	0.532	0.001
T-SOD (U mgprot-1)	215.55 ± 45.29^b^	282.53 ± 69.88^ab^	312.82 ± 38.95^a^	299.06 ± 45.07^a^	0.018	0.016	0.025
GSH-PX (U mgprot-1)	73.09 ± 12.65^b^	68.08 ± 12.39^b^	100.23 ± 16.29^a^	106.87 ± 15.93^a^	<0.001	<0.001	0.704
LDH (U gprot-1)	2164.66 ± 299.19^a^	2178.23 ± 162.79^a^	1926.79 ± 275.64^a^	1528.58 ± 190.57^b^	<0.001	<0.001	0.284
MDA (nmol mgprot-1)	0.94 ± 0.19^a^	0.59 ± 0.08^b^	0.39 ± 0.06^c^	0.40 ± 0.05^c^	<0.001	<0.001	<0.001

^a,b,c^Means with no common superscripts in the same row denote significant differences (*P* < 0.05). Results were the Mean ± SD (*n* = 6).

### 3.4 Expression of antioxidant genes in the ileum

As shown in Supplementary Figure 1, compared with the control group, the mRNA expressions of *Keap1* and *Nrf2* were significantly increased in the ileum samples of the TP treatment groups (*P* < 0.05). The TM and TH groups exhibited a significant increase in the expressions of *SOD1* and *SOD2* (*P* < 0.05). The TM group displayed a significant increase in the expression of *CAT* and *HO-1* mRNAs (*P* < 0.05).

### 3.5 Effect on the immunology indices of the serum and ileum samples

As shown in Table 8, compared with the control group, the serum contents of IgA and IgG in squabs in the TP treatment groups showed a significant increase (*P* < 0.05), while the IgM content was significantly increased in the TM and TH groups (*P* < 0.05).

**TABLE 8:** Effects of tea polyphenols on serum immune index of squabs.

Items	C group	TL group	TM group	TH group	ANOVA *P*-value	Linear *P*-value	Ouadratic *P*-value
IgA (μg mL-1)	60.42 ± 0.57^b^	67.82 ± 0.98^a^	68.97 ± 1.85^a^	72.57 ± 8.27^a^	0.001	<0.001	0.101
IgM (μg mL-1)	151.68 ± 3.15^b^	156.34 ± 2.42^b^	167.59 ± 4.06^a^	165.39 ± 7.46^a^	<0.001	<0.001	0.004
IgG (μg mL-1)	430.36 ± 12.23^b^	473.43 ± 10.07^a^	465.02 ± 17.98^a^	465.32 ± 14.97^a^	<0.001	0.004	0.001

^a,b^Means with no common superscripts in the same row denote significant differences (*P* < 0.05). Results were the Mean ± SD (*n* = 6). IgA, immunoglobulin A; IgM, immunoglobulin M; IgG, immunoglobulin G.

As listed in Table 9, the ileum contents of IL-1β and TNF-α in the TP treatment groups were significantly lower than those in the control group (*P* < 0.05), whereas the contents of IL-10 and IL-10/TNFα were significantly increased (*P* < 0.05). Also, the contents of sIgA and IL-2 were significantly increased in the ileum of the TM group (*P* < 0.05).

**TABLE 9:** Effects of tea polyphenols on ileal immune index of squabs.

Items	C group	TL group	TM group	TH group	ANOVA *P*-value	Linear *P*-value	Ouadratic *P*-value
sIgA (ng ml-1)	424.31 ± 26.59^b^	456.40 ± 23.34^ab^	499.12 ± 22.39^a^	459.61 ± 37.69^ab^	0.002	0.042	0.001
IL-1β (pg ml-1)	119.95 ± 2.90^a^	93.49 ± 2.57^c^	103.00 ± 4.04^b^	97.58 ± 4.22^bc^	<0.001	<0.001	<0.001
IL-2 (pg ml-1)	63.94 ± 2.42^b^	66.17 ± 1.69^ab^	67.78 ± 2.93^a^	65.14 ± 1.89^ab^	0.052	0.478	0.009
IL-10 (pg ml-1)	371.28 ± 0.53^b^	373.27 ± 0.12^a^	373.01 ± 0.24^a^	373.24 ± 0.51^a^	<0.001	<0.001	<0.001
TNF-α (pg ml-1)	222.65 ± 1.33^a^	196.01 ± 7.16^b^	200.08 ± 1.09^b^	196.72 ± 4.05^b^	<0.001	<0.001	<0.001
IL-10/TNF-α	1.67 ± 0.01^b^	1.91 ± 0.07^a^	1.86 ± 0.01^a^	1.9 ± 0.04^a^	<0.001	<0.001	<0.001

^a,b,c^Means with no common superscripts in the same row denote significant differences (*P* < 0.05). Results were the Mean ± SD (*n* = 6). sIgA, secretory IgA; IL-1β, interleukin-1β; IL-2, interleukin-2; IL-10, interleukin-10; TNF-α, tumor necrosis factor-α.

### 3.6 Effect of TPs on ileum morphology development

Concerning the effect of TPs on the ileum morphology of squabs, the villus height/crypt depth ratio was significantly higher in TP-treated squabs than in the control squabs (*P* < 0.05, Supplementary Figure 2D). The ileal villus height was significantly increased in the TL and TH groups (*P* < 0.05, Supplementary Figure 2B), whereas the ileal crypt depth was significantly decreased in the TM group (*P* < 0.05, Supplementary Figure 2C). These results indicated that dietary supplementation with TPs in the breeding pigeons did no significant damage to the ileal tissue morphology. Meanwhile, the ileal villi of squabs in the TM and TH groups were significantly thicker and more densely arranged than those of the control group (Supplementary Figure 2A), suggesting that TPs may protect the integrity of the ileum villi.

### 3.7 Effect of TPs on the expression of tight junction protein-related genes in the ileum

The mRNA expression levels of *Claudin-3*, *Occludin*, and *ZO-1* in the ileum of squabs in TP treatment groups were significantly higher than those in the control group (*P* < 0.05, Supplementary Figures 3B–D), while *Claudin-2* was significantly downregulated (*P* < 0.05, Supplementary Figure 3A).

### 3.8 Effect of TPs on digestive enzyme activity in the ileum

As listed in Table 10, the activities of α-amylase and lipase in the ileum of TP treatment groups were significantly decreased compared to the control group (*P* < 0.05), whereas the activity of trypsin was significantly increased in the ileum of TH group squabs (*P* < 0.05).

**TABLE 10:** Effect of tea polyphenols on digestive enzyme activity in ileal of squabs.

Items	C group	TL group	TM group	TH group	ANOVA *P*-value	Linear *P*-value	Ouadratic *P*-value
α-amylase (U mgprot-1)	0.26 ± 0.05^a^	0.17 ± 0.05^b^	0.15 ± 0.07^b^	0.17 ± 0.02^b^	0.004	0.019	0.003
Lipase (U gprot-1)	8.29 ± 1.40^a^	5.47 ± 1.21^b^	5.19 ± 1.25^b^	5.16 ± 0.73^b^	<0.001	0.001	0.003
Trypsi n (U mgprot-1)	99.06 ± 37.11^b^	106.16 ± 27.14^ab^	123.18 ± 21.25^ab^	151.17 ± 32.25^a^	0.032	0.004	0.799

^a,b^Means with no common superscripts in the same row denote significant differences (*P* < 0.05). Results were the Mean ± SD (*n* = 6).

### 3.9 Effect of TPs on microbial structure in the ileum

We next explored the effect of TPs on the composition of intestinal flora in squabs by 16S rRNA gene sequencing. As shown in Table 11, TP treatment groups showed a significant decrease in the Shannon index (*P* < 0.05) and an increase in the Simpson index (*P* < 0.05), while the Sobs, ACE, and Chao1 indices showed no significant difference compared to the control group (*P* > 0.05). The Venn diagram showed 60 common OTUs between the control (C) and the TP treatment groups. The C, TL, TM, and TH groups contained 100, 19, 114, and 19 unique OTUs, respectively (Supplementary Figure 4A). Principal Component Analysis (PCA) showed that the intestinal microflora structure in TP treatment groups significantly differed from that in the control group (*P* = 0.001, Supplementary Figure 4B). This indicated that TPs had a significant effect on the intestinal microflora structure of squabs. At the phylum level, Firmicutes, Proteobacteria, and Actinobacteria were the main phyla of the ileal microbiota; Firmicutes were dominant (Supplementary Figure 4C). At the genus level, the ileal microbiota of squabs was dominated by *Candidatus Arthromitus*, *Lactobacillus*, *Burkholderia-Caballeronia-Paraburkholderia*, *Aeriscardovia*, *Turicibacter*, *Bifidobacterium*, and *norank _ f _ Muribaculaceae*. *Candidatus Arthromitus*, and *Lactobacillus* genera (Supplementary Figure 4D). The significant difference test for comparison between the two groups was performed to screen the differentially expressed bacteria. Among the top 10 bacteria with relative abundance at the genus level, *Candidatus Arthromitus* and *Corynebacterium* were found to be enriched in TP treatment groups (*P* < 0.05), and *Lactobacillus*, *Aeriscardovia*, *Bifidobacterium*, and *norank_f_Muribaculaceae* were enriched in the control group (*P* < 0.05, Supplementary Figures 5A–C).

**TABLE 11:** Alpha diversity index analysis of tea polyphenols on intestinal flora of squabs.

Items	C group	TL group	TM group	TH group	ANOVA *P*-value	Linear *P*-value	Ouadratic *P*-value
Sobs	115.33 ± 75.37	54.00 ± 17.93	110.33 ± 68.40	55.83 ± 16.02	0.092	0.173	0.964
ACE	233.40 ± 92.25	187.35 ± 96.19	199.97 ± 98.04	125.94 ± 72.59	0.250	0.062	0.853
Chao1	171.68 ± 95.46	101.26 ± 41.81	166.40 ± 88.61	89.28 ± 34.77	0.117	0.132	0.808
Shannon	1.81 ± 0.48^a^	0.69 ± 0.50^b^	0.57 ± 0.25^b^	0.78 ± 0.36^b^	<0.001	0.002	<0.001
Simpson	0.30 ± 0.15^b^	0.70 ± 0.26^a^	0.78 ± 0.10^a^	0.67 ± 0.21^a^	0.001	0.009	0.001

^a,b^Means with no common superscripts in the same row denote significant differences (*P* < 0.05). Results were the Mean ± SD (*n* = 6).

Furthermore, network analysis was performed to understand the relationship between differently enriched microorganisms and antioxidant and inflammatory parameters in the top 10 bacteria at the genus level (Supplementary Figure 5D). *Corynebacterium* and *Aeriscardovia* belong to Actinobacteriota, and *Lactobacillus* belongs to Firmicutes. *Candidatus Arthromitus* belongs to the family Clostridiaceae (phylum Firmicutes). The abundance of *Corynebacterium* showed a significant positive correlation with ileal GSH-Px activity (*P* < 0.05), and the abundance of *Candidatus Arthromitus* was negatively correlated with the ileal contents of IL-1β and MDA (*P* < 0.05). The abundances of *Lactobacillus* and *Aeriscardovia* were positively correlated with ileal MDA content (*P* < 0.05), while sIgA showed the opposite trend (*P* < 0.05).

## 4 Discussion

In recent years, several studies have confirmed that TPs can improve the growth performance of animals. Xue et al. (2017) identified that EGCG can significantly increase the body weight, average daily gain, and average daily feed intake of broilers under heat stress. It also improves the antioxidant capacity of the body, enhances the growth performance of broilers, and alleviates the oxidative damage of broilers under heat stress. Erener et al. (2011) reported that green tea extract significantly increased body weight, feed efficiency, and carcass weight. Moreover, diets with green tea extract at 200 mg/kg were deemed suitable for the growth performance of broilers. Keyvan et al. (2018) showed that ration supplementation with Chinese green tea extract maintained broiler growth and improved feed utilization. Our results indicated that TPs had no significant effect on the weight and feed intake of squabs. However, a linear increase in the final weight and average daily gain of squabs was observed. This significantly increased the nest feed conversion ratio of squabs. On the one hand, TPs can promote the intestinal development of squabs and improve their ability to digest and absorb nutrients. On the other hand, TPs positively affect the growth performance of squabs by regulating intestinal microbial diversity and improving intestinal health (Williamson and Clifford, 2010; Li et al., 2019). The same has also been confirmed in our experiments.

Under normal conditions, the continuous production and elimination of free radicals are in a state of dynamic balance. When this balance is disrupted, it can cause oxidative stress and damage to cells, leading to various diseases. The health benefits of TPs are mainly attributed to their antioxidant properties and the ability of polyphenolic catechins to scavenge reactive oxygen (Ahmed et al., 2017). Through *in vitro* and *in vivo* experiments, TPs have been established as an ideal source of natural antioxidants (Truong and Jeong, 2022). Wu et al. (2014) confirmed that oolong tea powder increased the serum activity of SOD and decreased the MDA content in Cherry Valley meat ducks. Wang et al. (2019) reported that TPs alleviated the negative effects of high levels of molybdenum on liver function indices (such as elevated ALT and AST activities), antioxidant capacity, and production performance of 65-week-old laying hens. Song J. et al. (2019) reported that EGCG significantly increased the activities of GSH-Px, SOD, and CAT and decreased MDA content in the jejunum of Arbor Acres broiler chickens. This alleviated the intestinal oxidative damage caused by heat stress and improved the morphological development of broilers. Indeed, our study demonstrated that TPs can enhance the activity of antioxidant enzymes in both the serum and intestine of squabs. Furthermore, they were found to effectively inhibit the formation of lipid peroxides, thereby significantly improving the antioxidant capacity of the TP-treated squabs. This may be related to the special chemical structure of polyphenols. Aromatic phenolic compounds have multiple hydroxyl groups, making them good hydrogen or electron donors. These donors can neutralize free radicals and other reactive oxygen species (Zhang and Tsao, 2016).

Meanwhile, we conducted molecular-level investigations to study the effect of TPs on the Nrf2-ARE pathway. The results indicated that TPs could activate the Nrf2-ARE antioxidant pathway, inducing the expression of *Nrf2* and downstream antioxidant enzyme-related genes. This enhances the antioxidant capacity in the intestines of squabs. These findings are consistent with an increase in antioxidant enzyme activity in the serum and ileum of squabs, as well as the inhibition of oxidative damage. Wang et al. (2020) found that 165 mg/kg feed-supplemented EGCG significantly improved the gene expression and protein levels of P38MAPK, Nrf2, and HO-1 in the liver of 35-week-old Lohmann laying hens. This indicated that EGCG could enhance the antioxidant capacity of laying hens by activating the MAPK/Nrf2 signaling pathway. Song J. et al. (2019) showed that EGCG supplementation promoted the expression of *Nrf2* in the jejunum of broilers to improve their antioxidant capacity. Qi et al. (2017) showed that TPs reversed decreases in H_2_O_2_-elicited cell viability and mitochondrial dysfunction and upregulated the protein levels of Nrf2, Keap1, HO-1, and NQO1. *In vivo* experiments further confirmed that TPs upregulated the gene expressions and protein levels of HO-1 and NQO1 in mice liver, and effectively ameliorated H_2_O_2_ triggered oxidative stress. It can be observed that regardless of the body’s condition, whether in a healthy or stressed state, TPs can regulate the Nrf2-ARE pathway. This regulation initiates the expression of downstream antioxidant enzyme-related genes, thereby enhancing the body’s antioxidant capacity or alleviating oxidative damage.

Serum biochemical indicators can reflect animal health to a certain extent (Liu et al., 2023). Serum total protein, including albumin and globulin, reflects both nutritional status and protein metabolism (Liu et al., 2019). ALT indicates liver cell damage, while AST serves as a biomarker of mitochondrial damage (Sayed et al., 2022). When liver damage occurs, serum ALT and AST activities increase (Nyblom et al., 2004). Serum cholesterol and triglyceride levels indicate lipid absorption and metabolism (Ding et al., 2016). Wu et al. (2014) demonstrated that Oolong tea powder significantly reduced Cherry Valley ducks’ abdominal fat yield and serum triglyceride levels. It also improved fat deposition and blood lipid levels in ducks. Huang et al. (2015) showed that EGCG reduced serum TG content and AST activity in broilers. It also regulated the expression of lipid metabolism-related genes and key liver enzymes, substantially reducing abdominal fat deposition in broilers. Rizk et al. (2017) found that green tea reduced serum AST and ALT activities and the levels of total lipids, triglycerides, and total cholesterol in Sinai laying hens. Simultaneously, it increased serum total protein, albumin, and globulin levels, enhancing animal immunity. These findings are consistent with our results showing that dietary TPs could protect liver function and regulate lipid metabolism. This effect can be attributed to the lipid-lowering effects of TPs, involving reduced amylase and lipase activities, inhibited lipid absorption and fat accumulation, increased fatty acid oxidation, and enhanced fecal excretion (Uchiyama et al., 2011; Friedrich et al., 2012). Simultaneously, TPs significantly raised squab serum immunoglobulin levels. Immunoglobulins, as bioactive proteins, enhance nutrient uptake, stimulate growth, and regulate the immune system (Lönnerdal et al., 2017). Studies have reported that TPs can inhibit inflammatory cytokine production and promote immunoglobulin secretion (Liu et al., 2021; Ma et al., 2021).

The serum immunoglobulin-antigen interaction level reflects humoral immunity function (Sun et al., 2010). Song D. et al. (2019) discovered that supplementing Camellia oleifera seed extract and microencapsulated *Enterococcus faecalis* increased serum IgG and IgA levels in Hy-Line Brown laying hens. It also reduced total cholesterol, enhancing lipid and protein metabolism. Sha et al. (2020) found that TPs decreased serum IL-6, IL-1β, and TNF-α levels, inhibiting NF-κB pathway-mediated inflammatory responses. Liu et al. (2017) showed that orally administering 300 mg/kg TPs to male rats increased serum IL-10 levels and the IL-10/TNF-α ratio while reducing serum IL-1β, IL-6, and TNF-α levels, mitigating acute exhaustive exercise-induced inflammation. These results mirrored our findings of raised ileum sIgA and anti-inflammatory cytokine levels, and reduced pro-inflammatory cytokine levels. All of these improve squab intestinal immune barrier function, offering intestinal immune function protection.

Intestinal morphology is essential for nutrient digestion, absorption, and transport (Sansonetti, 2004). Intestinal villi, which are protrusions on the inner surface of the small intestinal wall, are formed by numerous folds. The crypt depth indicates the cell formation rate. The ratio of villi to crypt (VCR) is a crucial intestinal health and absorption efficiency indicator. Higher villus height, shallower crypt depth, and a larger villus-to-crypt ratio suggest enhanced intestinal absorption and secretion capabilities (Montagne et al., 2003; Paiva et al., 2014; Wu et al., 2018). Wei et al. (2021) observed that EGCG significantly increased mouse small intestine villus height and the villus-to-crypt ratio, and decreased crypt depth. It alleviated gastrointestinal tract damage induced by cyclophosphamide, restoring normal villus and crypt structures. Gallic acid, a natural phenolic compound, has been shown to increase broiler jejunum villus height and VCR, decrease crypt depth, and enhance nutrient absorption, thus improving growth performance (Samuel et al., 2017). Our results align with these studies, showing that TPs promoted ileum development in squabs to various degrees and improved nutrient absorption in their intestines.

Intestinal mucosal epithelial cells form a physical barrier through tight junctions, which prevent pathogens and macromolecules from passing through. The tight junction proteins regulate the permeability and polarity of cell layers, which are essential for innate immunity (Soini, 2011). Lu et al. (2021) found that polyphenol extracts increased the expressions of *Occludin, ZO-1*, *Claudin-1*, *Claudin-2*, and *Claudin-3* in the small intestine and promoted the integrity of the small intestinal barrier in mice. Zhang et al. (2019) showed that Chinese sweet leaf tea extract upregulated *ZO-1* and downregulated *Claudin-2* in the small intestine of mice, which effectively prevented lipopolysaccharide from entering the circulation system to induce systemic inflammation. The above results are similar to those in this study. Dietary TPs increase the levels of intestinal tight junction proteins, protecting the integrity of the intestinal barrier in squabs.

Our results showed that TPs significantly reduced the activities of lipase and α-amylase in the ileum of squabs and inhibited lipids and starch decomposition. This may regulate lipid metabolism in squabs (Tucci et al., 2010). Several studies have shown that the inhibition of various digestive enzymes, including pancreatic lipase and amylase by polyphenols, is highly correlated with their molecular structure (Surai, 2014; Sun et al., 2019). The results are similar to those in this study, where TPs reduced the serum contents of TC and TG in squabs. Simultaneously, TPs increased the activity of ileal trypsin, which promoted the digestion and absorption of nutrients such as protein in the intestine, consistent with the effect of TPs on the serum contents of TP and GLO.

Pennekamp et al. (2018) reported hump and U-shaped effects of diversity on overall ecosystem stability. When biodiversity is low, biodiversity can increase overall ecosystem stability and vice versa. The effect of diversity on ecosystem multifunctionality would also behump- or U-shaped effect if diversity has a positive effect on some functions and a negative effect on others. Our study demonstrated that feed supplementation with TPs altered the composition of the pigeon microbiota, resulting in a decrease in microbial community diversity. Nagendra (2002) showed that the Shannon diversity index responds most strongly to changes in the rarest species, while the Simpson index responds most strongly to changes in the proportional abundance of the most common species. We speculated that a decline in microbial diversity might be due to a reduction in the proportion of particular taxa, as the richness indices (Chao 1 and ACE) exhibited no significant effect. In recent years, dietary modulation of the gut microflora has been an effective strategy for improving gut health and productivity in poultry. Dietary polyphenols have gained substantial attention for their potent antioxidant properties, which can regulate gut flora and improve intestinal health by generating more bioactive metabolites (Iqbal et al., 2020). Hanski et al. (2012) demonstrated that environmental biodiversity, host commensal microbiota, and the immune system are all complex systems with many components (i.e., species and molecules) interacting. Overall, the impact of the diversity of animal microbiota is complex and requires a combination of factors to be considered. Our results showed that Firmicutes were the dominant bacteria at the phylum level in the ileum flora of control and experimental squabs. Notably, Firmicutes are known for their strong fermentation ability and production of SCFAs and lipid metabolism. Similar results were reported by Sun et al. (2022). Our results showed that under normal conditions (in the C group), the intestinal tract of squabs was colonized by a large number of beneficial bacteria (*Lactobacillus*, *Aeriscardovia*, and *Bifidobacterium*), which promoted intestinal peristalsis to improve digestion and absorption of nutrients (Ma et al., 2020). Meanwhile, the relative abundance of *Lactobacillus* and *Aeriscardovia* decreased when the diets were supplemented with TPs, positively correlated with MDA, and negatively correlated with sIgA. We hypothesized that this phenomenon might be related to the increase in the abundance of *Candidatus Arthromitus* and *Corynebacterium*. The abundance of *Candidatus Arthromitus* was negatively correlated with IL-1β and MDA content, and the abundance of *Corynebacterium* was positively correlated with GSH-Px activity. *Candidatus Arthromitus* specifically modulates the host immune response, including Th17 cell differentiation, induction of gut IgA plasma cells, and intestinal IgA secretion. Its reduced abundance may lead to an imbalance in the intestinal immune system and a range of inflammatory responses (Ma et al., 2020; Zhang B. et al., 2020). *Corynebacterium* has a strong metabolic capacity to metabolize a wide range of sugars, organic acids, and alcohols and is used for the large-scale production of amino acids (Eikmanns and Blombach, 2014). Probiotics were found to be an essential factor in the growth and development of squabs (Ding et al., 2020). Li et al. (2019) reported that TPs improved host health through the regulation of intestinal microbiota. Zhang et al. (2021) reported that plant polyphenols hold the homeostasis of the intestinal microenvironment, stimulate the growth of symbiotic beneficial microbiota, and inhibit pathogenic strains (e.g., *Clostridium perfringens* and *Bacteroides* spp.) but have little effect on symbiotic anaerobes (e.g., *Bifidobacterium* spp. and *Lactobacillus* spp.). Zhang L. et al. (2020) showed that TPs could improve intestinal flora by increasing the activity of antioxidant enzymes and expression of tight junction proteins in the ileum and attenuating inflammation and oxidative stress markers in mice. In our study, TPs-fed squabs showed improved health status without any adverse effects, as indicated by physiological indices (Supplementary Figure 6). Changes in the abundance of intestinal flora in squabs were significantly correlated with intestinal antioxidant and immune-related parameters, indicating that TPs can also promote intestinal health in squabs under healthy conditions.

## 5 Conclusion

This study showed that dietary supplementation with TPs in breeder pigeons may improve growth performance, serum biochemical indices, antioxidants, and immunity, and maintain the intestinal health of squabs by enhancing intestinal function. Under experimental conditions, the addition of 200,400 mg/kg of tea polyphenols to the diet was more effective.

## Data availability statement

The datasets presented in this study can be found in online repositories. The names of the repository/repositories and accession number(s) can be found below: https://www.ncbi.nlm.nih.gov/, PRJNA1047616.

## Ethics statement

The animal study was approved by the Animal Care Committee of Zhongkai University of Agriculture and Engineering, Guangdong, China (Ethical approval number: 20200717). The study was conducted in accordance with the local legislation and institutional requirements.

## Author contributions

AC: Conceptualization, Data curation, Formal analysis, Methodology, Software, Writing – original draft. TM: Data curation, Validation, Writing – original draft. YZ: Data curation, Validation, Writing – original draft. SD: Investigation, Writing – original draft. SZ: Investigation, Writing – original draft. ZF: Investigation, Writing – original draft. YH: Project administration, Supervision, Writing – review and editing. JF: Formal analysis, Methodology, Supervision, Writing – review and editing.
