# Supplementary material for: Effect of tea polyphenols supplement on growth performance, antioxidation, and gut microbiota in squabs
Source: Front Microbiol. 2024 Jan 15;14:1329036. doi: 10.3389/fmicb.2023.1329036 (PMC10822925; doi:10.3389/fmicb.2023.1329036)
Supplement: Supplementary file 1 [file Data_Sheet_1.pdf]

frontiers

Effect of tea polyphenols supplement on growth performance, antioxidation, and gut microbiota in squabs

Ailing Chen[1, 2], Tingting Ma[1, 2], Yajing Zhong[1, 2], Shan Deng[1, 2], Shaoping Zhu[3], Zhiqi Fu[1, 2], Yanhua Huang[*1, 2, 3] and Jing Fu[*1, 2]

[1]Innovative Institute of Animal Healthy Breeding, Zhongkai University of Agriculture and Engineering, Guangzhou, Guangdong 510225, China.

[2]College of Animal Science and Technology, Zhongkai University of Agriculture and Engineering, Guangzhou, Guangdong 510225, China.

[3]Guangdong Laboratory for Lingnan Modern Agriculture, Guangzhou, Guangdong 510642, China.

*** Correspondence:**

Yanhua Huang*

huangyh111@126.com

Jing Fu*

fujing1999@163.com

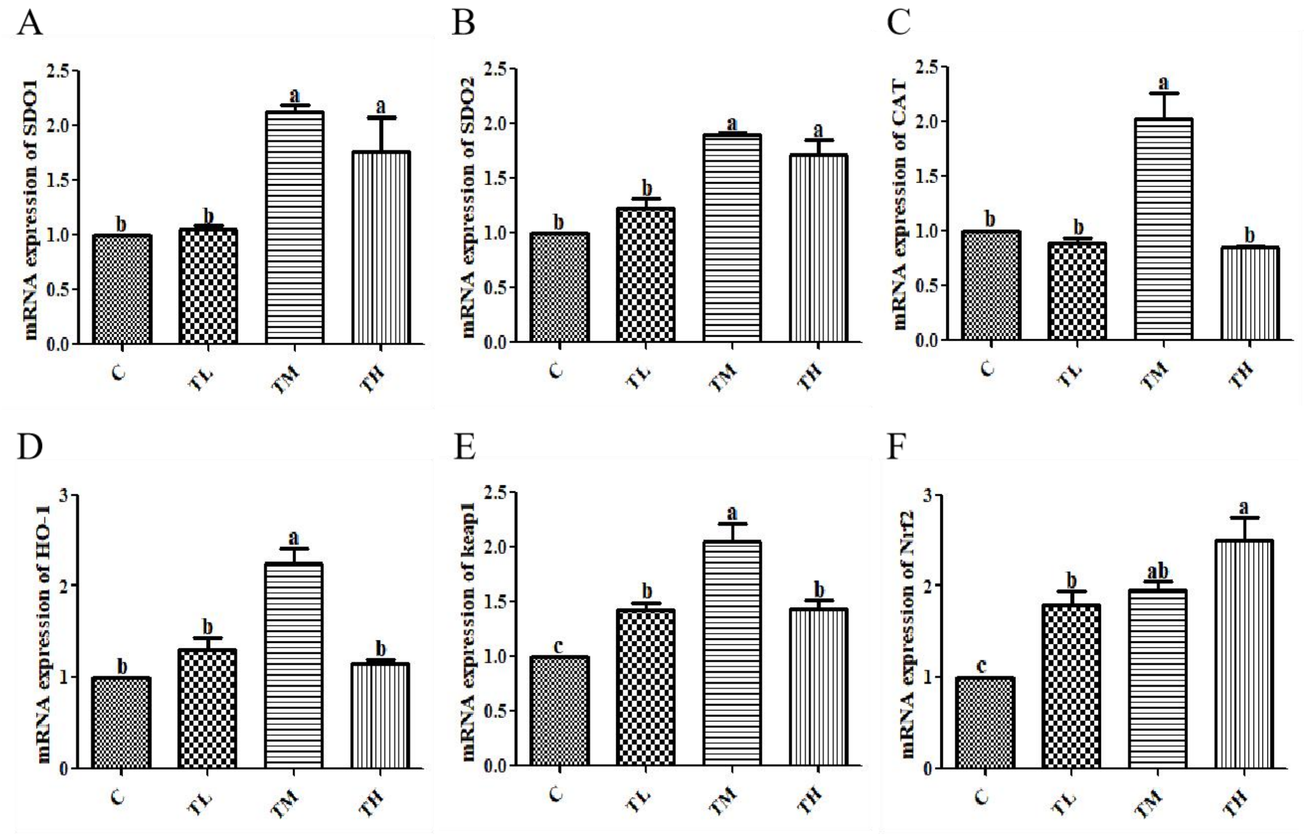

Supplementary Figure S1. Effects of tea polyphenols on expression of antioxidant genes in the ileum of squabs. (A) SOD1, superoxide dismutase1. (B) SOD2, superoxide dismutase2. (C) CAT,catalase. (D) HO-1, Heme Oxygenase-1. (E) Keap1, Kelch-like ECH-associated protein 1. (F) Nrf2, NF-E2-related factor2. ^{a,b,c} Means with no common superscripts in the same row denote significant differences (P<0.05). Results were the Mean±SD (n=6).

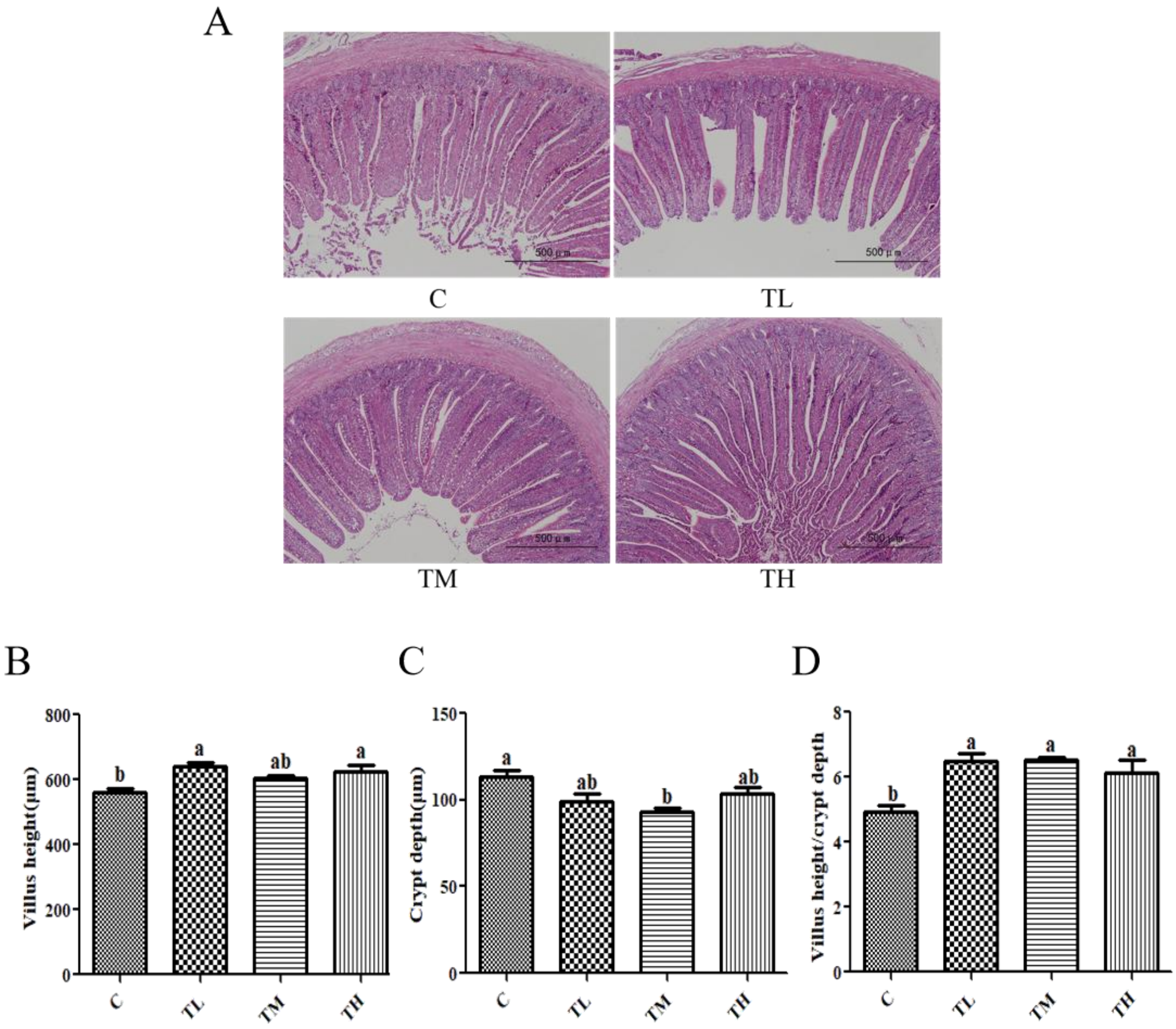

Supplementary Figure S2 Effects of tea polyphenols on ileal morphology of 28-day-old squabs. (A) Representative haematoxylin and eosin-stained morphology in the ileum. Pictures were taken at ×100 magnification. (B) Ileal villus height. (C) Ileal crypt depth. (D) Ileal villus height/crypt depth. [a,b] Means with no common superscripts in the same row denote significant differences ($P<0.05$). Results were the Mean±SD (n=6).

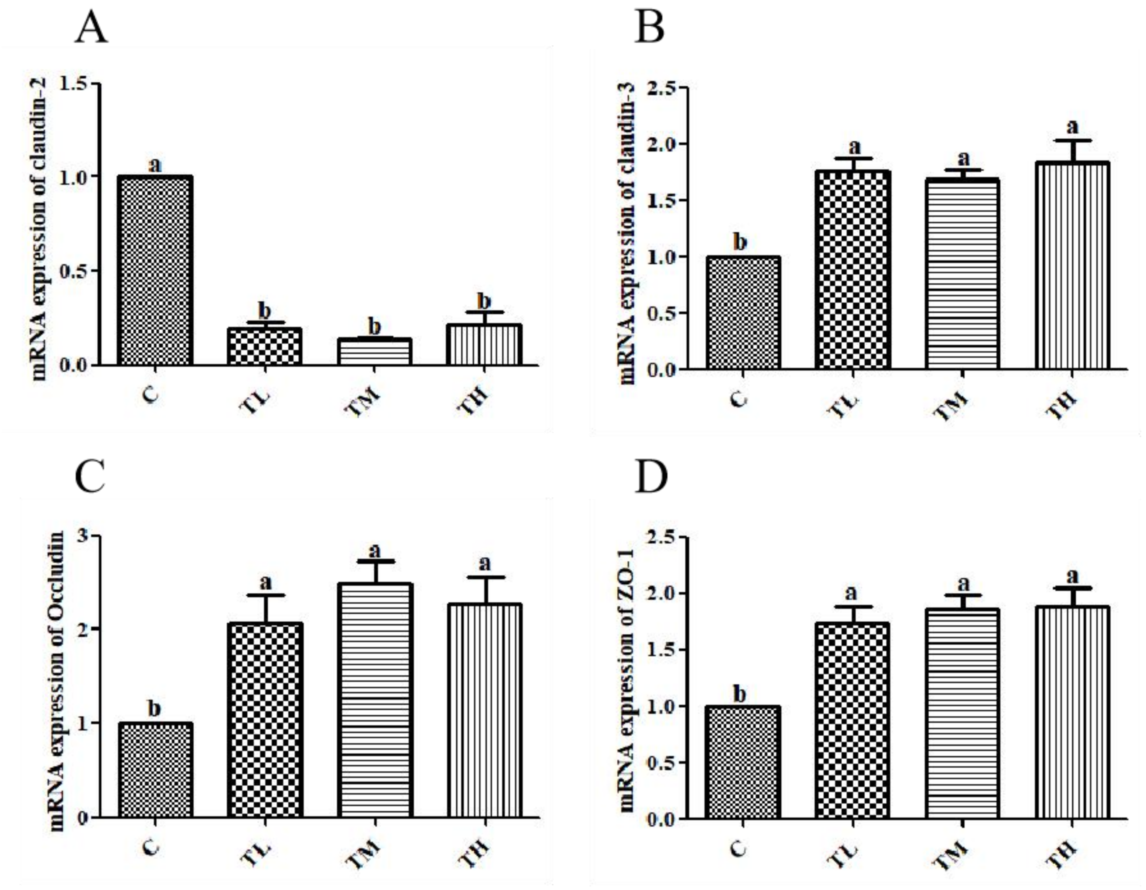

Supplementary Figure S3 Effect of tea polyphenols on the expression of tight junction protein related genes in the ileum of squabs. (A) Claudin-2. (B) Claudin-3. (C) Occludin. (D) ZO-1. [a,b] Means with no common superscripts in the same row denote significant differences (*P*<0.05). Results were the Mean±SD (n=6).

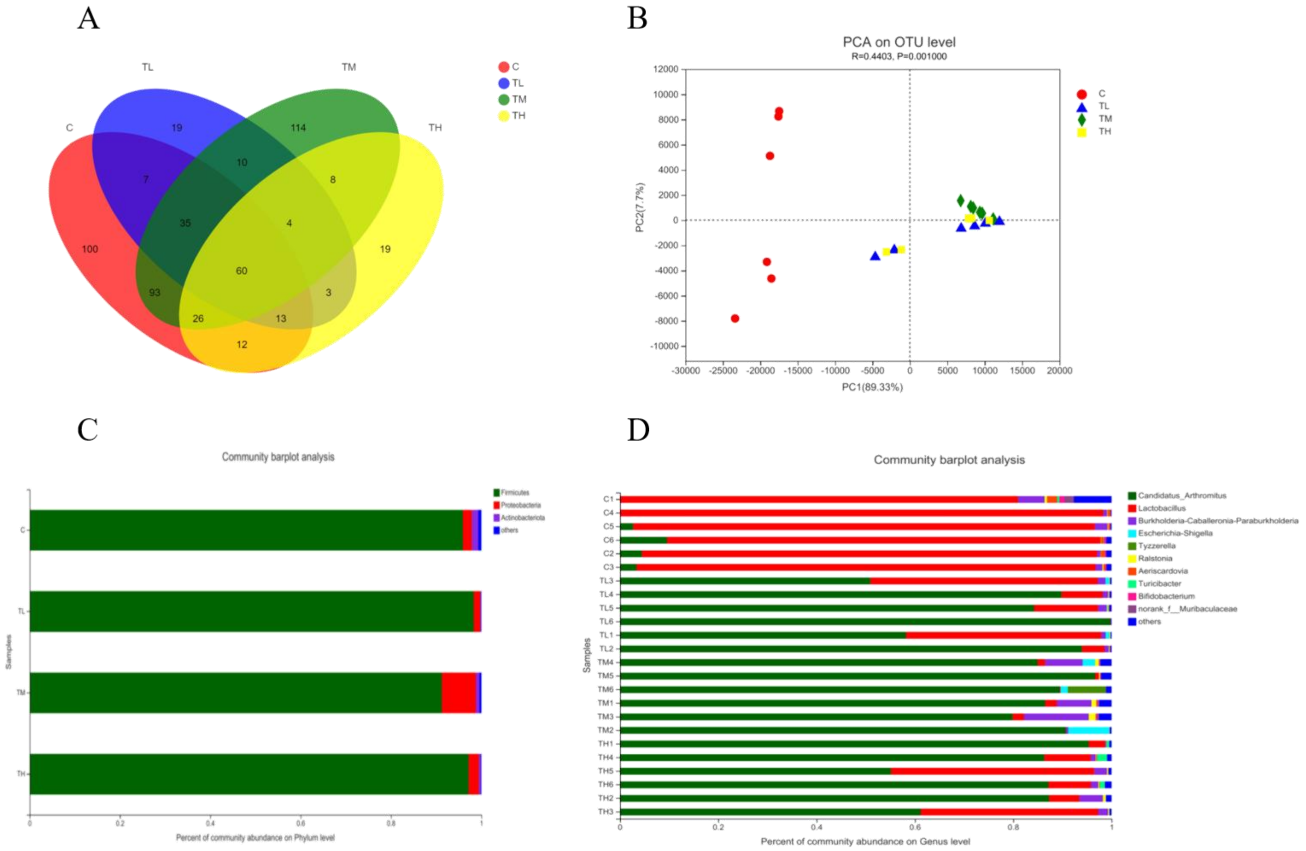

Supplementary Figure S4 Effects of tea polyphenols on ileal microbial composition in squabs. Wayne diagram analysis of microorganisms in the ileum of squabs on d 28 (A). PCA analysis of ileum flora in squabs on d 28 (B, Anosim analysis, P=0.001<0.05). Species relative abundance of ileal flora in squabs at phylum level (C) and genus level (D).

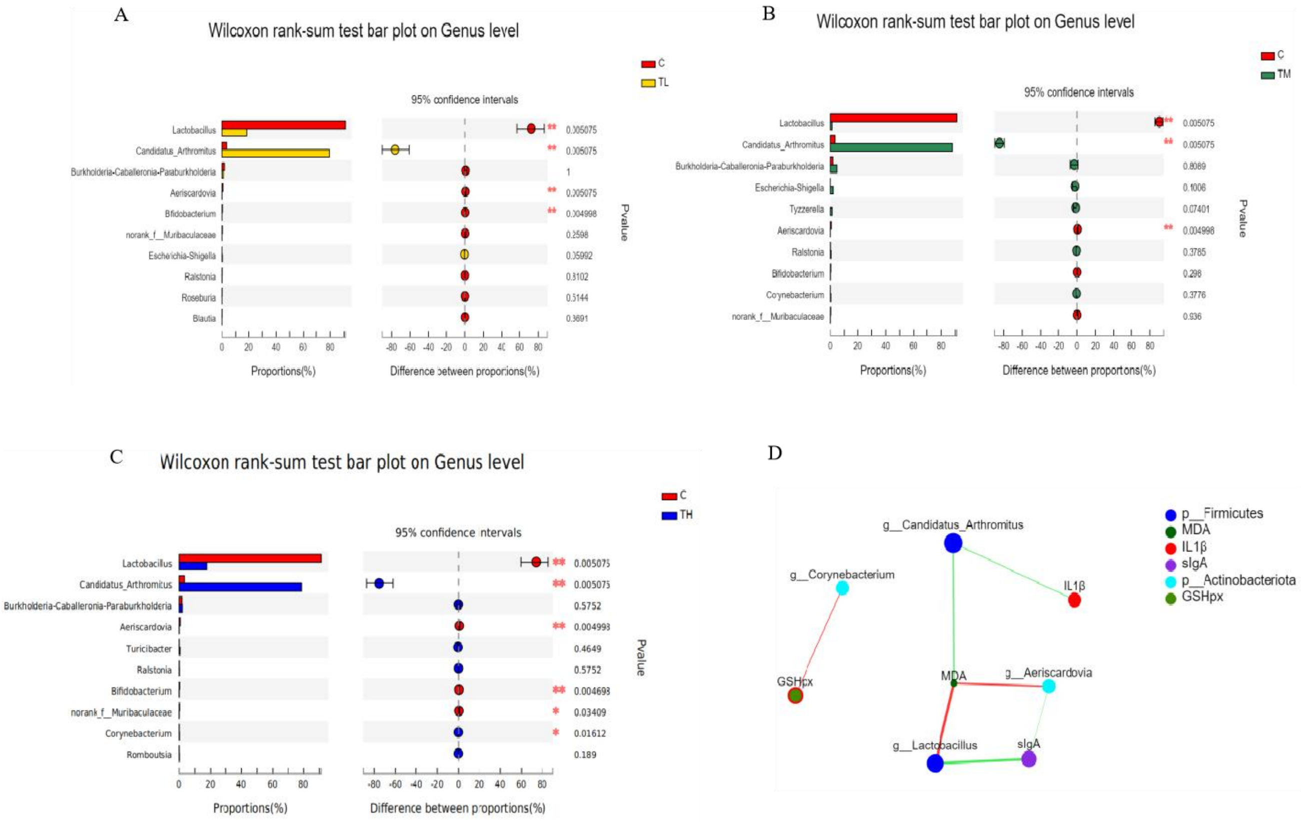

Supplementary Figure S5 Species difference analysis of ileal flora in squabs at genus level (A, C group and TL group; B, C group and TM group; C, C group and TL group) and Network analysis (D). The red lines and the green lines indicate positive correlation and negative correlation respectively.

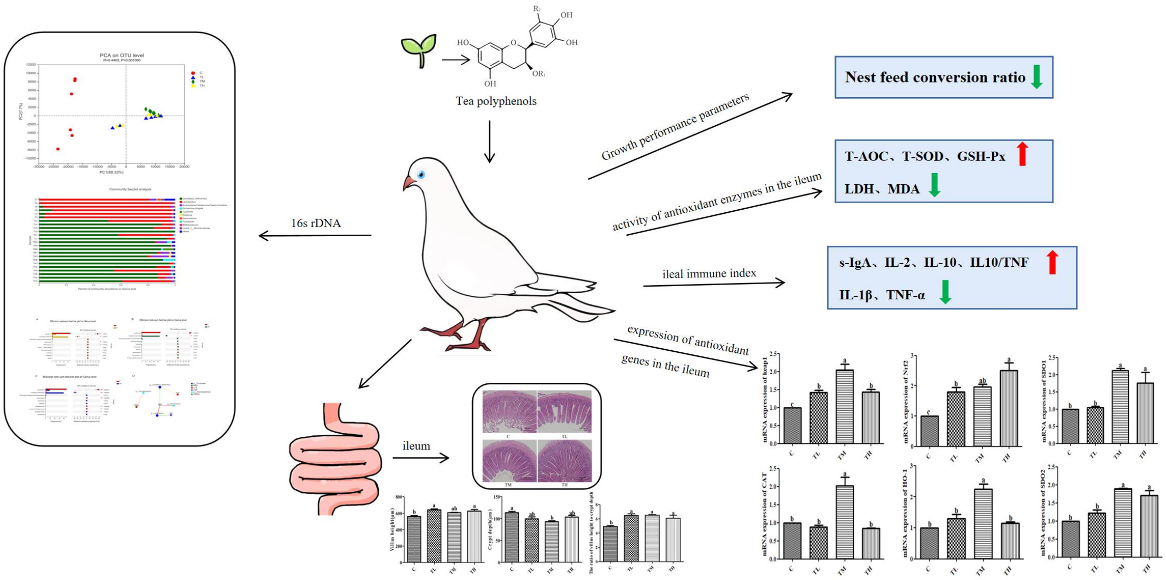

Supplementary Figure S6 Antioxidant, immune and intestinal barrier effects of tea polyphenols in squabs.
